# Electrocatalytic and Solar‐Driven CO_2_ Reduction to CO with a Molecular Manganese Catalyst Immobilized on Mesoporous TiO_2_

**DOI:** 10.1002/anie.201601038

**Published:** 2016-04-25

**Authors:** Timothy E. Rosser, Christopher D. Windle, Erwin Reisner

**Affiliations:** ^1^ Christian Doppler Laboratory for Sustainable SynGas ChemistryDepartment of ChemistryUniversity of CambridgeLensfield RoadCambridgeCB2 1EWUK

**Keywords:** carbon dioxide, electrocatalysis, hybrid materials, manganese, reduction

## Abstract

Electrocatalytic CO_2_ reduction to CO was achieved with a novel Mn complex, *fac*‐[MnBr(4,4′‐bis(phosphonic acid)‐2,2′‐bipyridine)(CO)_3_] (**MnP**), immobilized on a mesoporous TiO_2_ electrode. A benchmark turnover number of 112±17 was attained with these TiO_2_|**MnP** electrodes after 2 h electrolysis. Post‐catalysis IR spectroscopy demonstrated that the molecular structure of the **MnP** catalyst was retained. UV/vis spectroscopy confirmed that an active Mn–Mn dimer was formed during catalysis on the TiO_2_ electrode, showing the dynamic formation of a catalytically active dimer on an electrode surface. Finally, we combined the light‐protected TiO_2_|**MnP** cathode with a CdS‐sensitized photoanode to enable solar‐light‐driven CO_2_ reduction with the light‐sensitive **MnP** catalyst.

The reduction of CO_2_ to CO is viewed as a potentially lucrative and renewable source of a key chemical feedstock, as well as a strategy to reduce rising atmospheric CO_2_ levels. Electrocatalysis by molecular transition‐metal complexes is a viable means of achieving this transformation, typically offering excellent tunability^1^ and selectivity^2^ as well as providing opportunities to study the catalytic mechanism.^3^ Alternatives based on inexpensive solid‐state materials usually offer less well‐defined catalytic centers that prevent a detailed understanding of the catalytic mechanism.^4^

Immobilization of such molecular catalysts on electrode surfaces makes efficient use of the active metal centers and therefore enables a true appraisal of properties, such as the turnover number (TON).^5^ However, in most cases reported to date, molecular catalysts were deposited on carbon5c, 6 and Pt‐based^7^ electrodes. These offer low transparency to visible light, and only in very few cases have the surface‐bound catalytic intermediates been characterized spectroscopically in situ.2c, 8 Bimolecular reaction mechanisms, in which active dimers form during catalysis, have not been observed on electrode surfaces, and it has been thought that such mechanisms would be impeded by immobilization of a monomeric pre‐catalyst.5b, 9

First‐row transition‐metal complexes based on [MnBr(CO)_3_(L)] (L=bipyridine and derivatives) have emerged in recent years as promising electrocatalysts for CO_2_ reduction owing to their high selectivity and low overpotential for catalysis.^10^ They also contain only Earth‐abundant elements, which is a significant advantage over analogous Re‐based catalysts.7b, 8, 11 The low overpotential is a direct consequence of the bimolecular reaction mechanism, whereby a Mn^0^—Mn^0^ dimer is formed after the first reduction of the homogeneous molecular catalyst, which then reduces CO_2_ to CO (L=4,4′‐dimethyl‐2,2′‐bipyridine).10a However, the maximum TONs achieved by this class of complex for electrocatalytic CO production are 34 after 18 h,10a and 36 after 6 h.^12^ Mn catalysts have been integrated onto electrodes in polymer films, such as Nafion, where they reached a TON of 14 based on the total amount of catalyst used.^13^ From electrochemical measurements it was proposed that the Mn^0^–Mn^0^ dimer forms in the polymer matrix, although this was not spectroscopically verified. Preliminary studies of an electro‐polymerized pyrrole‐based Mn catalyst deposited on silicon nanowires have also suggested photoelectrochemical (PEC) CO_2_ reduction, based on cyclic voltammetry (CV) results.^14^

Herein, we present a novel Mn^I^ CO_2_ reduction electrocatalyst with a phosphonate functionality (**MnP**, Scheme 1) that allows anchoring and direct wiring between the catalytic center and a metal oxide surface,^15^ as has been achieved for an analogous phosphonate‐modified Re complex.^16^ We employ a mesoporous TiO_2_ electrode, because it offers 1) long‐term stability and conductivity under reducing conditions,^17^ 2) a three‐dimensional morphology for high catalyst loading and to facilitate close inter‐molecular interactions, and 3) transparency for spectroelectrochemical characterization of catalytic intermediates.^18^ The electrochemical investigations establish the heterogenized **MnP** as the best‐performing Mn electrocatalyst to date, which was enabled by a dynamic TiO_2_|**MnP** interface and dimerization of the immobilized Mn catalyst. Finally, we present the first example of CO_2_ reduction by a Mn catalyst driven by full UV/Vis solar‐spectrum irradiation, circumventing the typical photo‐instability13b, 19 of these compounds by combining the TiO_2_|**MnP** hybrid cathode in the dark with a CdS‐sensitized photoanode.

**Scheme 1:**
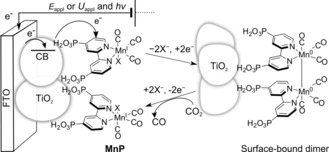
Schematic representation and proposed mechanism for CO_2_ reduction by TiO_2_|**MnP** (X=Br^−^ in the isolated compound).

**MnP** (Scheme 1) was synthesized by coordination of 4,4′‐bis(phosphonic acid)‐2,2′‐bipyridine to pentacarbonyl manganese(I) bromide in ethanol under N_2_ while protected from light. The product was isolated as an orange solid in 63 % yield and characterized by CHNP microanalysis, ^31^P‐NMR spectroscopy, high‐resolution mass spectrometry, and infrared (IR) spectroscopy ($\bar{\nu }$_CO_=2030, 1946, and 1930 cm^−1^, Figure 1 a), which confirmed a *fac‐*Mn tricarbonyl species.^19^ Full synthetic and characterization details can be found in the Supporting Information. **MnP** was insoluble in CH_3_CN and therefore characterized by CV in DMF (Figure S1 in the Supporting Information). A catalytic wave at *E*_onset_=−1.8 V versus Fc^+^/Fc (Fc=[(η‐C_5_H_5_)_2_Fe]) was observed when H_2_O was added and the cell was purged with CO_2_. The presence of water in the electrolyte solution is known to significantly increase electrocatalytic CO_2_ reduction activity, by allowing the Mn–Mn dimer to directly react with CO_2_.10a

**Figure 1:**
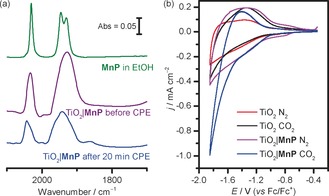
a) Solution FTIR of **MnP** and ex situ ATR‐FTIR spectra of TiO_2_|**MnP** before and after controlled‐potential electrolysis (CPE) for 20 min at *E*_appl_=−1.7 V versus Fc^+^/Fc. b) CV scans of TiO_2_ and TiO_2_|**MnP** (geometrical surface area = 1.0 cm^2^) under N_2_ and CO_2_. Conditions: CH_3_CN/H_2_O (19/1), 0.1 m Bu_4_NBF_4_, *ν*=100 mV s^−1^, Ag/AgCl reference electrode (RE), Pt counter electrode (CE), room temperature.

Mesoporous TiO_2_ electrodes were prepared by a doctor‐blading procedure, applying a suspension of commercial P25 TiO_2_ nanoparticles (anatase/rutile (8/2) mixture, average particle size 21 nm) to a fluorine‐doped tin oxide (FTO) coated glass electrode, and further experimental details can be found in the Supporting Information. Scanning electron microscopy (SEM) on the resultant electrode revealed a mesoporous film with a thickness of approximately 6 μm (Figure S2 a). Loading of the catalyst onto the TiO_2_ electrode was achieved by drop‐casting a methanol solution of **MnP**, resulting in 34 nmol Mn per cm^2^ of geometrical surface area. The presence of IR bands at $\bar{\nu }$_CO_=2032 and 1928 cm^−1^ in the attenuated total reflectance Fourier transform infrared (ATR‐FTIR) spectrum confirmed the presence of **MnP** on the electrode (TiO_2_|**MnP**; Figure 1 a). Immobilization and electronic communication of the **MnP** with a metal oxide was confirmed by adsorbing **MnP** on conducting and mesoporous tin‐doped indium oxide (ITO) electrodes instead (film thickness approximately 7 μm, see Figure S2 b and Supporting Information for experimental details). CV with ITO|**MnP** in anhydrous CH_3_CN (1.0 m Bu_4_NBF_4_) displayed a reversible wave at *E*=−1.6 V versus Fc^+^/Fc, assigned to the reduction of Mn^I^ to Mn^0^_._ The peak current was linearly dependent on the scan rate, indicative of an immobilized species in good electronic communication with the electrode (Figure S3).

TiO_2_ becomes conductive at potentials more negative than the conduction band (CB), thus the CV of TiO_2_|**MnP** can be employed to study electrocatalytic CO_2_ reduction. The CV scan of a bare (Mn‐free) TiO_2_ electrode in CH_3_CN/H_2_O (19/1, 0.1 m Bu_4_NBF_4_) shows the filling and emptying of the conduction band of TiO_2_ (Figure 1 b), as confirmed by the increase in absorbance in the *λ*=600–850 nm region of the electronic spectrum at an applied potential, *E*_appl_, of −1.8 V versus Fc^+^/Fc (Figure S4).17b,^20^ Comparable CV features are observed with a bare TiO_2_ electrode under CO_2_ or TiO_2_|**MnP** under N_2_. However, TiO_2_|**MnP** purged with CO_2_ showed an increased current with an onset of *E*=−1.6 V versus Fc^+^/Fc, indicative of electrocatalytic CO_2_ reduction by the heterogenized **MnP** catalyst (Figure 1 b). Furthermore, the ratio of cathodic to anodic charge in the forward and reverse CV scans increased from approximately 1:1 to 4:1 by changing TiO_2_ to TiO_2_|**MnP** under CO_2_, suggesting that conduction‐band electrons of TiO_2_ are consumed by the Mn catalyst on the CV timescale and are therefore unavailable for discharging during the anodic scan.

The increased current arising from TiO_2_|**MnP** under CO_2_ was confirmed as being the result of the reduction of CO_2_ to CO by controlled‐potential electrolysis (CPE). Figure 2 a shows the gaseous products formed when TiO_2_|**MnP** electrodes were held at *E*_appl_=−1.7 V versus Fc^+^/Fc in the dark under CO_2_, and monitored by gas chromatography (GC). After 2 h, an average of 1.10±0.25 C was passed, with the production of 3.75±0.56 μmol CO, corresponding to a Faradaic efficiency (FE) of 67±5 %. The FE for H_2_ production was 12.4±1.4 %, and the formation of formate was not detectable by ion chromatography. The TON_CO_ of 112±17 was calculated based on the amount of **MnP** drop‐cast onto the electrode, and is thus a lower limit since it assumes all **MnP** remains bound and active throughout CPE. This is the highest TON_CO_ based on the total amount of catalyst used for a Mn catalyst in CO production, and was achieved at a low overpotential (*η*) of approximately 0.42 V, calculated using a standard potential for CO_2_ reduction to CO (*E* ^0′^(CO_2_/CO)) of −1.28 V versus Fc^+^/Fc in these conditions.^21^ This is one of the lowest overpotentials observed for a transition‐metal‐based catalyst in non‐aqueous solution,1a, 2a, 22 matched only by a modified Fe‐porphyrin in homogeneous DMF solution (*η*=0.41 V)21a and a Mn catalyst that achieved a TON_co_ of 36 after 6 h (*η*=0.35 V).^12^

**Figure 2:**
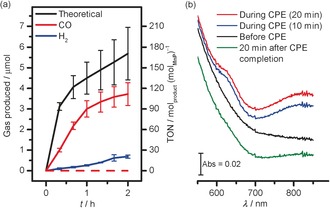
a) Electrocatalytic CO production by TiO_2_|**MnP** (solid lines) performed with *E*_appl_=−1.7 V versus Fc^+^/Fc for 2 h, and theoretical maximum based on charge passed in the CPE. Dashed lines show no CO production in the absence of **MnP** or CO_2_. b) In situ UV/Vis spectroelectrochemistry of TiO_2_|**MnP** under CPE at *E*_appl_=−1.7 V versus Fc^+^/Fc for 20 min. Lower wavelength data are not shown because of strong scattering from the mesoporous TiO_2_. CPE conditions: CH_3_CN/H_2_O (19/1, 0.1 m Bu_4_NBF_4_, Pt CE, Ag/AgCl RE) under CO_2_ at room temperature.

TiO_2_|**MnP** exhibited good CO selectivity, with a CO:H_2_ ratio of approximately 12:1 after 1 h CPE, although this ratio was reduced to 5.4:1 after 2 h, presumably a result of desorption or degradation of the Mn catalyst during the second hour of electrolysis. In the absence of either CO_2_ or the Mn catalyst (Figures S5 a and S5 b), no CO was produced. H_2_ production by bare TiO_2_ was 1.91±0.31 μmol after 2 h, compared to 1.43±0.22 μmol for TiO_2_|**MnP** with a surface coverage of 22 nmol cm^−2^ and 0.69±0.08 μmol with a coverage of 34 nmol cm^−2^ (see Figure 2 a, Figure S5, and Table S1). Increasing amounts of **MnP** on TiO_2_ therefore suppress H_2_ in favor of CO production, suggesting that H_2_ production by TiO_2_|**MnP** may originate from unmodified areas of the TiO_2_ rather than the catalyst itself.

IR and UV/Vis spectroscopies confirmed the molecular nature of **MnP** during catalysis on TiO_2_. Figure 1 a shows an ATR‐FTIR spectrum of TiO_2_|**MnP** taken after CPE for 20 min (*Q*=0.37 C, approximate TON_CO_=34), revealing peaks at $\bar{\nu }$_CO_=2042 and 1943 cm^−1^. These vibrational CO stretches closely match the spectrum of the as‐prepared electrode, with a slight shift explained by exchange of coordinated Br^−^ for a solvent molecule, and therefore demonstrate that the molecular structure of the catalyst remains largely unchanged during catalytic turnover. Deactivation of the Mn catalyst to a material that is no longer molecular would be unlikely to give high CO selectivity, corroborating Figure 2 a.

The UV/Vis spectra of TiO_2_|**MnP** before, during, and after 20 min CPE with *E*_appl_=−1.7 V versus Fc^+^/Fc are shown in Figure 2 b. During CPE, bands at 630 and 820 nm were observed, which are assigned to the formation of an Mn–Mn dimer by comparison to similar peaks formed during homogeneous CPE of the unmodified [MnBr(bpy)(CO)_3_] (Table S2 for assignment).1a, 2b We excluded the formation of the mononuclear doubly reduced **MnP** anion, analogues of which are also known to reduce CO_2_ when dimer formation is impeded,1b, 2c due to the lack of a strong peak at approximately 548 nm as found in an analogous Mn compound in THF3a (difference spectrum in Figure S6). After CPE for 20 min, the TiO_2_|**MnP** was left under CO_2_ without an applied potential, and the peaks resulting from the dimer were lost (Figure 2 b). This was corroborated by the IR spectrum in Figure 1 a, which indicated mainly the presence of the Mn^I^ monomer, but with a small peak at 1865 cm^−1^ and a broadening of the peak at 1943 cm^−1^, assigned to a small amount of remaining dimer.3b These data are consistent with the mechanism shown in Scheme 1, with the formation of a steady‐state concentration of the catalytically active Mn–Mn dimer. This intermediate then reacts with CO_2_ before it can be identified ex situ, reforming the Mn^I^ monomer as detected in the IR spectrum.

Immobilization of **MnP** on mesoporous TiO_2_ creates a high local concentration of Mn^0^ under reducing conditions at the electrode surface. Phosphonic acid modified molecules, such as **MnP**, display some lability when bound to TiO_2_,^23^ and phosphate buffer has been used to displace anchored catalysts from TiO_2_ particles, demonstrating a dynamic interaction.^24^ We propose that the high activity and low overpotential of this system is due to either temporary desorption of the catalyst, followed by dimerization and re‐anchoring within mesoporous TiO_2_, or the high local concentration of **MnP** placing the metal centers in an environment where they are predisposed to dimerization upon reduction.

Manganese carbonyl compounds, such as **MnP**, show instability under illumination,^19^ and tend to undergo photolysis and release CO ligands.^25^ Consequently, the few reports of Mn‐based CO_2_ reduction photocatalysis use monochromatic or narrowly filtered light to prevent decomposition of the catalyst.^14^, 25a, 26 This photo‐instability was observed for TiO_2_|**MnP**, which displayed a significantly lower CO production of 0.39±0.16 μmol (12±3 % FE) when CPE was performed under UV‐filtered 1 sun illumination (*λ*>420 nm to avoid TiO_2_ band‐gap excitation in this experiment) at −1.7 V versus Fc^+^/Fc for 2 h (Figure S7). The significant H_2_ production (1.74±0.6 μmol, 59±8 % FE) is consistent with degradation of **MnP** and possibly the formation of a catalytically active Mn deposit. Therefore, TiO_2_|**MnP** cannot be used directly in a CO_2_ reducing photocathode that efficiently absorbs sunlight and exposes the catalyst to irradiation.

An alternative strategy to drive CO_2_ reduction using full solar‐spectrum irradiation was implemented, integrating **MnP** into a photoelectrochemical circuit with a photoanode, wired to TiO_2_|**MnP**, which was kept in the dark. CdS‐sensitized ZnO nanosheet electrodes were prepared following a reported procedure (SEM in Figure S8 a),^27^ which absorb a broad spectrum of light below 530 nm according to the electronic spectrum shown in Figure S8b. These ZnO|CdS electrodes gave an anodic photocurrent in the presence of triethanolamine (TEOA) as a hole scavenger with an onset of −1.65 V versus Fc^+^/Fc, a potential at which TiO_2_|**MnP** gives a cathodic current from CO_2_ reduction (Figure 3 a). The linear‐sweep voltammetry (LSV) scan of a two‐electrode, two‐compartment PEC cell comprising a CdS|ZnO photoanode and a TiO_2_|**MnP** cathode (kept in the dark) in Figure 3 b shows a small photocurrent at zero bias, which increased as a bias potential (*U*_appl_) was applied. To confirm that CO was produced, we performed CPE in a two‐electrode configuration in CH_3_CN/H_2_O electrolyte solution (19/1, 0.1 m Bu_4_NBF_4_, 0.1 m TEOA, purged with CO_2_). An applied potential of 0.6 V for 1 h passed a charge of 0.26 C, and 0.36±0.07 μmol of CO (26 % FE, 2.6:1 CO:H_2_ ratio, TON_CO_=11, Figure S9) was measured. The lower CO production performance compared to the three‐electrode electrocatalytic system could be due to the potentially disruptive presence of TEOA in the electrolyte solution, the lower charge passed and the different potential at the cathode. Nevertheless, this is the first example of full spectrum solar‐light driven CO_2_ reduction with a Mn catalyst.

**Figure 3:**
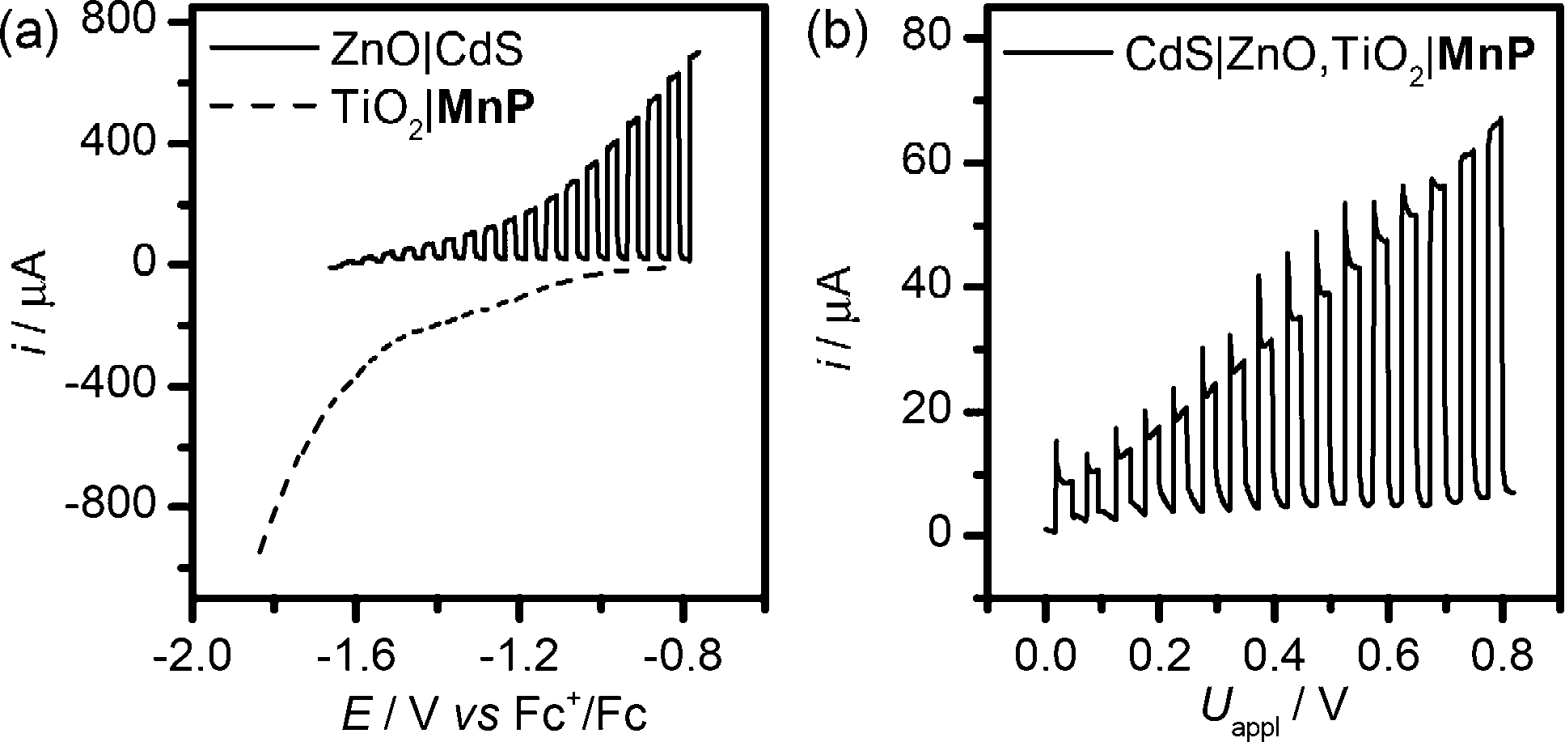
a) Three‐electrode LSV scans of ZnO|CdS and TiO_2_|**MnP** (Ag/AgCl RE, Pt CE). b) LSV scans of ZnO|CdS and TiO_2_|**MnP** in a two‐electrode configuration. Conditions: CH_3_CN/H_2_O (19/1, 0.1 m Bu_4_NBF_4_, 0.1 m TEOA (except three‐electrode TiO_2_|**MnP**), purged with CO_2_), simulated solar irradiation (AM 1.5 G, 100 mW cm^−2^), TiO_2_|**MnP** kept in the dark, room temperature.

In conclusion, we have presented **MnP** as a novel Mn‐based CO_2_ reduction catalyst that allows immobilization onto a mesoporous TiO_2_ electrode with its phosphonic acid anchoring groups. The TiO_2_|**MnP** cathode achieved efficient CO_2_ reduction to CO, reaching an unprecedented TON_CO_ of 112±17 at an overpotential of 0.42 V after 2 h CPE. During electrocatalytic CO_2_ reduction, a Mn–Mn dimer was formed, which is an important catalytic intermediate in homogeneous solution. This is, to our knowledge, the first observation of the dynamic formation of active catalytic dimers on a surface, providing a strategy for retaining homogeneous reaction mechanisms whilst also gaining the advantages of heterogeneous catalysis. Finally, we utilized the CO_2_ reduction activity of TiO_2_|**MnP** at a low overpotential to assemble a PEC cell with a CdS‐sensitized photoanode, demonstrating that Mn catalysts can be used in solar‐driven CO_2_ reduction in spite of their photo‐instability. This work represents an advance in moving molecular CO_2_ reduction electrocatalysis towards a full artificial photosynthetic system. This was achieved through the immobilization of the catalyst, attainment of a high TON at low overpotential, and implementation of a PEC cell.

## Supporting information

As a service to our authors and readers, this journal provides supporting information supplied by the authors. Such materials are peer reviewed and may be re‐organized for online delivery, but are not copy‐edited or typeset. Technical support issues arising from supporting information (other than missing files) should be addressed to the authors.

Supplementary
