# Supplementary material for: Electrocatalytic and Solar‐Driven CO2 Reduction to CO with a Molecular Manganese Catalyst Immobilized on Mesoporous TiO2
Source: Angew Chem Int Ed Engl. 2016 Apr 25;55(26):7388–92. doi: 10.1002/anie.201601038 (PMC5074277; doi:10.1002/anie.201601038)
Supplement: Supplementary file 1 — Supplementary [file ANIE-55-7388-s001.pdf]

Angewandte Chemie
Eine Zeitschrift der Gesellschaft Deutscher Chemiker

Supporting Information

Electrocatalytic and Solar-Driven CO_2 Reduction to CO with a Molecular Manganese Catalyst Immobilized on Mesoporous TiO_2

*Timothy E. Rosser+, Christopher D. Windle+, and Erwin Reisner**

anie_201601038_sm_miscellaneous_information.pdf

Experimental Section

Synthetic procedures

Chemicals were obtained from commercial suppliers and used without further purification, unless otherwise stated. Bu_4NBF_4 was either purchased as electrochemical grade or recrystallized from H_2O/EtOH (9/1) and dried in vacuo at 80 °C overnight before use. 4,4'-bis(phosphonic acid)-2,2'-bipyridine was prepared according to a literature procedure.[1] All solvents were dried before use. TiO_2 nanoparticles (P25, anatase/rutile (8/2) mixture, average particle size 21 nm) were a gift from Evonik Industries. Indium-tin oxide (ITO) nanopowder (<50 nm particle size) was obtained from Sigma-Aldrich.

***fac*-[Mn(Br)(4,4'-bis(phosphonic acid)-2,2'-bipyridine)(CO)$_3$] (MnP).** A Teflon screw cap Schlenk tube was charged with 4,4'-bis(phosphonic acid)-2,2'-bipyridine (142 mg, 450 µmol) and [MnBr(CO)$_5$] (123 mg, 450 µmol). Dry EtOH was added (approx. 15 mL) to the stirred starting materials via a syringe. The mixture was heated at reflux in the dark and the reaction progress followed by solution IR spectroscopy. The reaction showed complete conversion after approximately 2.5 h. The Schlenk tube was placed in the freezer to initiate precipitation. Addition of dry Et_2O with a syringe caused further precipitation of the product. The reaction mixture was filtered off and the bright orange solid was dried under reduced pressure. Yield: 152 mg (63%). NMR spectroscopy showed paramagnetic character. ^{31}P NMR (DMF-d$_7$): 9.27 (s, broad). HR-ESI-MS, –ve: *m/z* = 534.7510 ([M-H$^+$]$^-$, 60%); $C_{13}H_9BrN_2O_9P_2Mn$ requires 534.8347. 449.5436 ([M-H$^+$-(CO)$_3$]$^-$, 100%); $C_{10}H_9BrN_2O_6P_2Mn$ requires 449.9796. FT-IR ($\bar{\nu}$ / cm^{-1} in EtOH): 2030 (s), 1946 (s), 1930 (s) (CO); (ATR of solid): 2031, 1918 (br) (CO); 1156 (P=O). Anal Calcd for $C_{13}H_{10}BrN_2O_9P_2Mn$: C, 29.18; H, 1.88; N, 5.24; P, 11.58. Found: C, 29.01; H, 2.17; N, 5.38; P, 11.48.

Preparation of mesoporous metal oxide electrodes. Glass slides coated with tin-doped indium oxide (ITO) or fluorine-doped tin oxide (FTO) of dimensions 3 × 1 cm^2 were cleaned by heating at 70 °C in a 5:1:1 (vol:vol:vol) solution of H_2O:H_2O_2(10 M):NH_4OH(conc.) for 30 min, followed by rinsing with H_2O and drying at 180 °C for 1 h prior to the deposition of the mesoporous metal oxide film. Suspensions of ITO (20% by weight of ITO in a 5 M acetic acid solution in ethanol) and TiO_2 nanoparticles (100 mg TiO_2 and 50 mg poly(ethylene glycol in approximately 0.5 mL ethanol) were applied to the transparent conducting oxide-coated glass slides (ITO on ITO and TiO_2 on FTO-coated glass) using the doctor blading method using a Scotch tape mask with aperture dimensions of either a 6 mm diameter circle (for cyclic voltammetry) or a 0.7 cm × 1.5 cm rectangle (for (photo)electrolysis). The slides were then annealed at 450 °C for 0.5 h (mesoporous TiO_2) or at 400 °C for 1 h (mesoporous ITO). The mesoporous metal oxide electrodes were cleaned with ammonia/hydrogen peroxide and dried and/or using a BioForce UV/ozone cleaner. The electrodes were characterized by scanning electron microscopy (SEM), which revealed mesoporous films of approximate thickness 7 µm (ITO) and 6 µm (TiO_2).

Immobilization of MnP on mesoporous metal oxide electrodes. Modification was achieved by drop-casting 27 µL of a 1.3 mM methanol solution of **MnP** onto 1.0 cm^2 TiO_2 electrodes corresponding to a surface coverage of 34 nmol (geom. cm)$^{-2}$, in the dark in a N_2-filled glovebox, followed by drying for 15 min under N_2. For ITO, 20 nmol (geom. cm)$^{-2}$ was drop-cast onto 0.28 cm^2 electrodes.

Preparation of ZnO|CdS electrode. CdS-sensitized ZnO electrodes were prepared as previously described.[2] Briefly, we prepared ZnO by chemical bath deposition (CBD) from a solution containing $Zn(NO_3)_2$ and urea at 90 °C, followed by annealing and in situ formation of CdS quantum dots by CBD from a solution of $CdCl_2$ and thioacetamide at room temperature. SEM revealed nanosheets (ZnO) sensitized with CdS particles.

Electrochemical methods

Electrochemical measurements were performed on Ivium Compactstat or PalmSens Emstat potentiostats. Where stated (for three-electrode experiments), a Pt counter electrode (CE) and an Ag/AgCl reference electrode (RE) were used. The Ag/AgCl RE comprised a AgCl-coated Ag wire in the same electrolyte solution, separated from the solution by a Vycor frit, and was used to prevent leakage of Ag^+ into the electrolyte solution. Ferrocene was used as internal standard at the end of each three-electrode experiment. For cyclic voltammetry, a one compartment electrochemical cell was used, and the solution was not stirred. For controlled potential electrolysis (CPE), a custom-designed airtight two-compartment cell with a glass frit separating the counter electrode from the working and reference electrodes, was employed, and the solutions in both compartments were stirred. All experiments were performed at room temperature.

The electrolyte solution was purged with CO_2 or N_2 containing 2% CH_4 as an internal standard for gas chromatography (GC) where necessary. The purge gas was saturated with solvent using a bubbler to minimise evaporation of the test solutions. The Faradaic efficiency was calculated from the amount of CO and H_2 accumulated in the headspace (as measured by GC) and the charge passed through the external circuit. All CPE samples were also tested by ion chromatography, although formate was never observed above the detection limit. Where solar illumination was required, a Newport solar light simulator was used, with an intensity of 100 mW cm^{-2}, and AM1.5G filter and <420 nm cut-off filters used as required.

Physical measurements

^{31}P NMR spectra were recorded on a Bruker 400 MHz spectrometer. ^{31}P NMR spectra were referenced to an external standard (85% H_3PO_4 in D_2O). UV−vis spectra were recorded on a Varian Cary 50 UV−vis spectrophotometer. High-resolution electrospray ionization mass spectra (HR-ESI-MS) were recorded on a Quattro LC spectrometer, and the theoretical and experimental isotope distributions were compared. FTIR spectra were recorded on a Thermo Scientific Nicolet iS50 FT-IR spectrometer in attenuated total reflectance (ATR) or transmission modes. GC was performed on an Agilent 7890A instrument using CH_4 (2% in CO_2 or N_2) as an internal standard. H_2 was analysed using a HP-5 column (0.32 mm diameter) at 45 °C and N_2 carrier gas with a flow rate of approximately 3 mL min^{-1}. CO was analysed using a HP-PLOT/Q column (0.53 mm diameter) attached to a HP-5 column (0.32 mm diameter). The GC oven temperature was kept constant at 45 °C, Helium was used as carrier gas at an approximate flow rate of 2 mL min^{-1} and a thermal conductivity detector (TCD) was used.[3] SEM was performed on an FEI Philips XL30 sFEG instrument.

UV/vis absorption spectra of ZnO|CdS were recorded on an Edinburgh Instruments FS5 spectrofluorometer equipped with an integrating sphere. Synchronous scans ($\lambda_{ex} = \lambda_{em}$) were run for ZnO|CdS and FTO, and the absorption spectra generated by comparison with the sphere background using the Fluoracle software supplied with the FS5 instrument.

Supporting Tables

Table S1. Summary of electrocatalytic CO production

Electrode	Conditions[a]	*n*(CO) / μmol (Faradaic Efficiency)	TON_{CO}	*n*(H_2) / μmol (Faradaic Efficiency)
TiO_2|**MnP**		3.75±0.56 (67±5%)	112±17	0.69±0.08 (12.4±1.4%)
TiO_2|**MnP**	Purged with N_2	0	0	0.07±0.03 (7±6%)
TiO_2	No **MnP**	0	n/a	1.91±0.31 (41±7%)
TiO_2|**MnP**	22 nmol **MnP**	2.16±0.22 (37±4%)	96±8	1.43±0.22 (24±4%)
TiO_2|**MnP**	1 Sun[b]	0.39±0.17 (12±3%)	12±5	1.74±0.6 (59±8%)

[a]Conditions (unless otherwise stated): CPE in CH_3CN/H_2O (19/1, 0.1 M Bu_4NBF_4) at E_{appl} = –1.7 V *vs* Fc^+/Fc for 2 h. 33.6 nmol **MnP** on 1.0 cm^2 TiO_2, purged CO_2, Ag/AgCl reference electrode, Pt counter electrode. [b]1 Sun = AM1.5G, 100 mW cm^{-2}.

Table S2. UV/vis and IR Spectroscopic data for dissolved $[MnBr(bpy)(CO)_3]$ and TiO_2|**MnP**

Species	λ_{max} / nm	Ref	ν(CO) / cm^{-1}	ref
$[MnBr(CO)_3(bpy)]$	336 (sh), 416	[4]	2023 (s), 1935 (m), 1914 (m)	[5]
$[Mn(CO)_3(bpy)]_2$	404, 445, 538 (sh), 638, 817	[4]	1975 (m), 1963 (w), 1936 (s), 1886 (m), 1866 (m)	[5]
TiO_2|**MnP** before CPE	-[a]	This work	2033, 1927	This work
TiO_2|**MnP** after 20 min CPE	-[a] , 630[b], 820[b]	This work	2042[c], 1976(sh)[b], 1962 (sh)[b], 1942[c], 1861[b]	This work

[a]Scattering prevents the observation of bands below 550 nm (see Figure 2b main text). [b]Assigned to Mn-Mn dimer. [c]Assigned to Mn monomer.

Supporting Figures

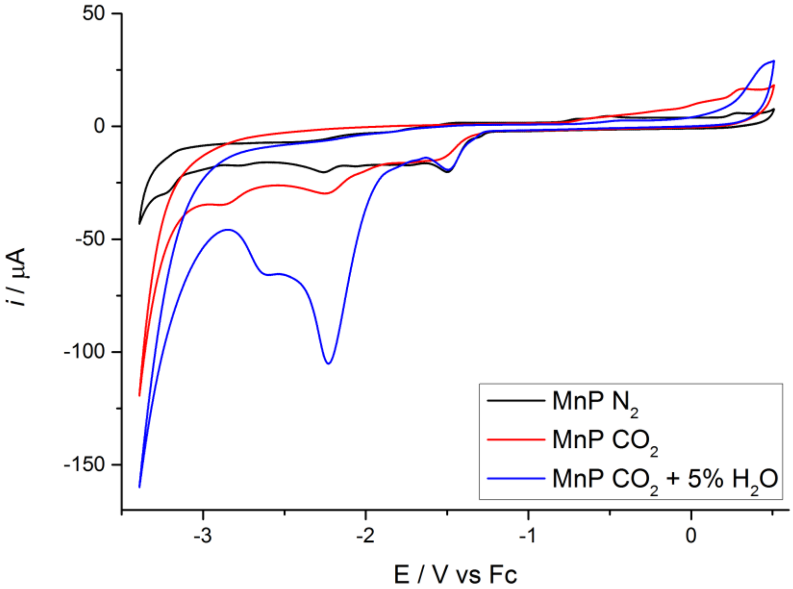

Figure S1. CV scans of **MnP** (1 mM) in DMF solution (0.1 M Bu_4NBF_4) at room temperature with added water where stated (5%). ν = 100 mV s^{-1}, Ag/AgCl RE, Pt CE, purged with CO_2 or N_2 as stated.

(a)

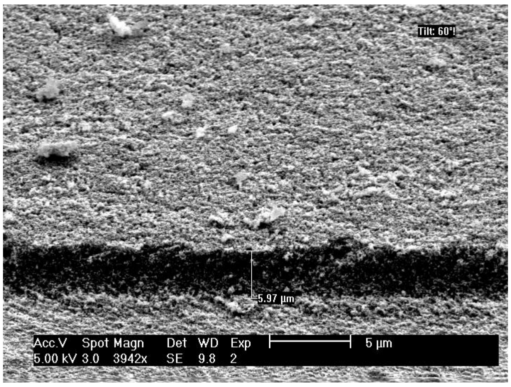

(b)

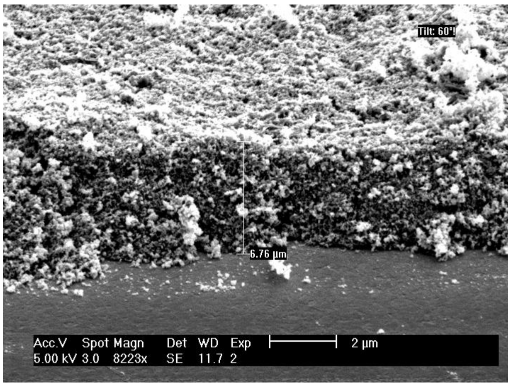

Figure S2. Cross-sectional SEM images of (a) mesoporous TiO_2 (approx. 6 μm thick) on FTO-coated glass and (b) mesoporous ITO (approx. 7 μm thick) on ITO-coated glass.

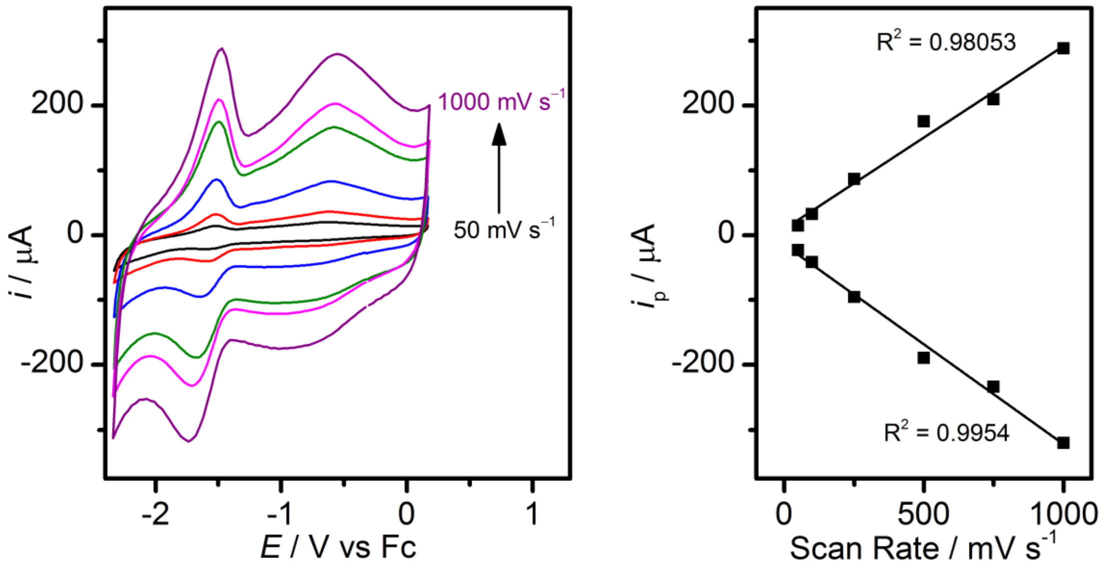

Figure S3. CV scans (left) and scan rate dependence (right) of **MnP** on ITO electrodes (prepared as described in experimental section) at scan rates of 50, 100, 250, 500, 750 and 1000 mV s^{-1}. Conditions: dry MeCN (1.0 M Bu_4NBF_4, purged with N_2), **MnP** loading 20 nmol cm^{-2}, Ag/AgCl RE, Pt CE. Peak current (i_p) taken for the Mn(I)→Mn(0) couple at –1.6 V vs Fc^+/Fc.

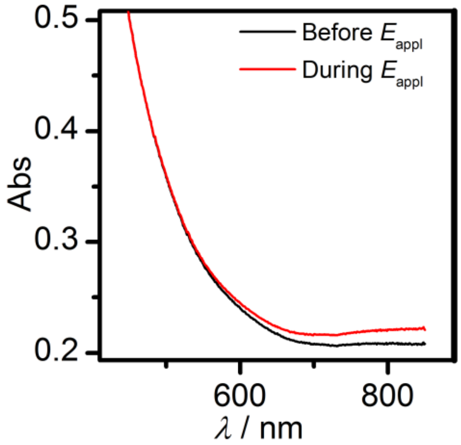

Figure S4 In-situ UV/vis spectroelectrochemistry of unmodified TiO_2 before and during application of E_{appl} = –1.7 V *vs* Fc^+/Fc for 20 min. Conditions: CH_3CN/H_2O (19/1, 0.1 M Bu_4NBF_4), Pt CE, Ag/AgCl RE, one compartment electrochemical cell at room temperature.

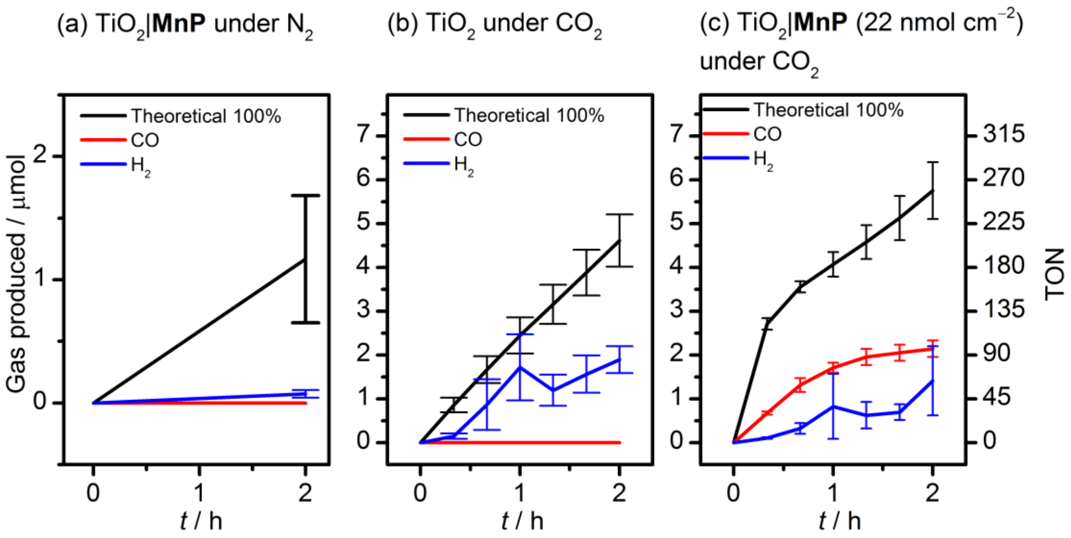

Figure S5. CPE by (a) TiO_2|**MnP** under N_2, (b) unmodified TiO_2 under CO_2 and (c) TiO_2|**MnP** under CO_2 at a lower surface coverage (22 nmol cm^{-2}). CPE conditions: CH_3CN/H_2O (19/1, 0.1 M Bu_4NBF_4) at E_{appl} = –1.7 V *vs* Fc^+/Fc, Pt CE, Ag/AgCl RE at room temperature.

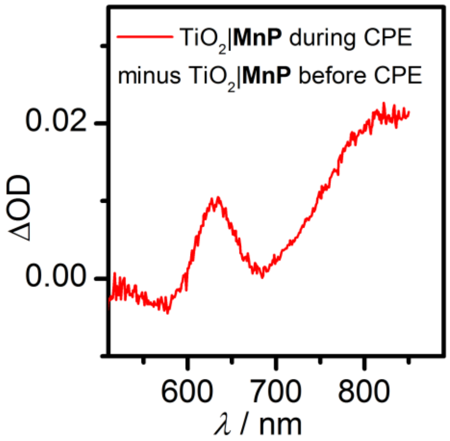

Figure S6. UV/vis spectrum of TiO_2|**MnP** after CPE for 20 min with a background spectrum of TiO_2|**MnP** before CPE subtracted, to reveal the peak positions. CPE conditions: CH_3CN/H_2O (19/1, 0.1 M Bu_4NBF_4) at E_{appl} = –1.7 V vs Fc^+/Fc for 20 mins, Ag/AgCl reference elctrode, Pt counter electrode at room temperature.

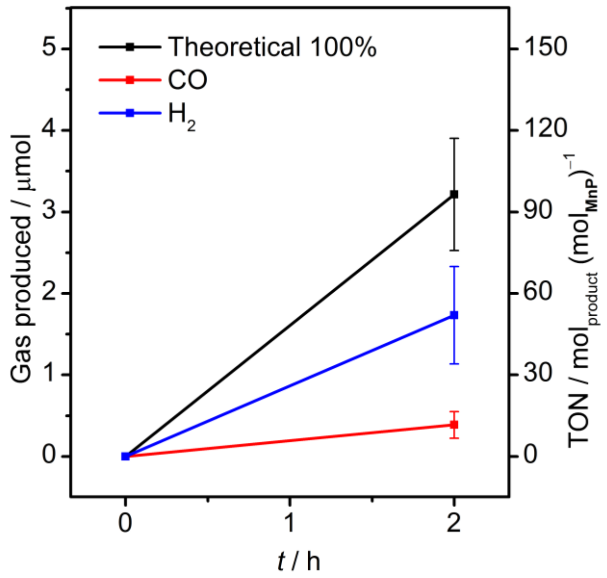

Figure S7. CPE with TiO_2|**MnP** under CO_2 under visible light irradiation (λ > 420 nm to avoid band gap excitation, AM1.5G, 100 mW cm^{-2}). CPE conditions: CH_3CN/H_2O (19/1, 0.1 M Bu_4NBF_4) at E_{appl} = –1.7 V *vs* Fc^+/Fc, Ag/AgCl reference electrode, Pt counter electrode at room temperature.

(a)

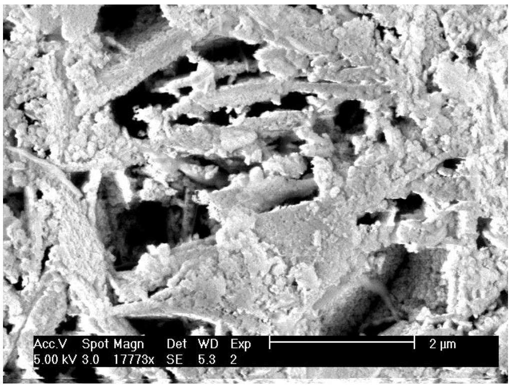

(b)

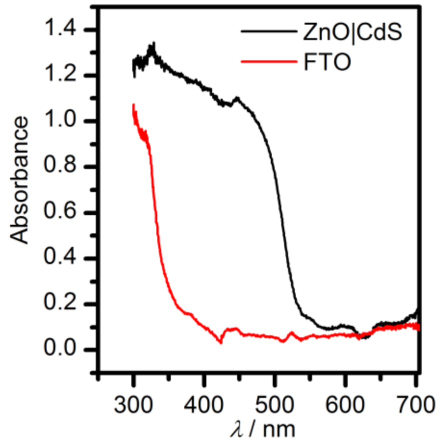

Figure S8 (a) SEM of CdS|ZnO. (b) UV/vis absorption spectra of ZnO|CdS and FTO-coated glass.

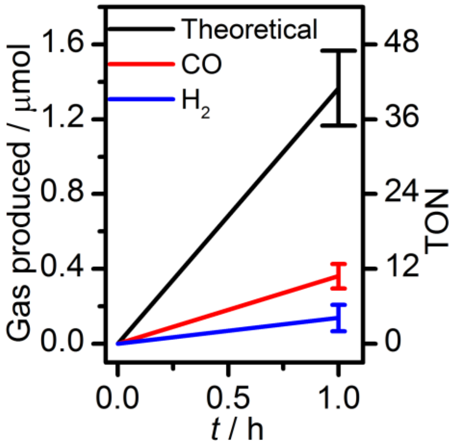

Figure S9. Photoelectrochemical CO production from a TiO_2|**MnP** cathode and a ZnO|CdS photoanode in a two electrode configuration. Conditions: CH_3CN/H_2O (19/1, 0.1 M Bu_4NBF_4, 0.1 M TEOA, purged with CO_2), simulated solar irradiation (AM1.5G, 100 mW cm^{-2}), TiO_2|**MnP** kept in the dark, U_{appl} = 0.6 V at room temperature.

Supporting References

[1] V. Penicaud, F. Odobel, B. Bujoli, *Tetrahedron Lett.* **1998**, *39*, 3689–3692.

[2] C.-Y. Lin, D. Mersch, D. A. Jefferson, E. Reisner, *Chem. Sci.* **2014**, *5*, 4906–4913.

[3] M. F. Kuehnel, D. W. Wakerley, K. L. Orchard, E. Reisner, *Angew. Chem. Int. Ed.* **2015**, *54*, 9627–9631.

[4] M. Bourrez, F. Molton, S. Chardon-Noblat, A. Deronzier, *Angew. Chem. Int. Ed.* **2011**, *50*, 9903–9906.

[5] F. Hartl, T. Mahabiersing, P. Le Floch, F. Mathey, L. Ricard, P. Rosa, S. Zális, *Inorg. Chem.* **2003**, *42*, 4442–4455.
